# Editorial: Ion Channel Signalling in Cancer: From Molecular Mechanisms to Therapeutics

**DOI:** 10.3389/fphar.2021.711593

**Published:** 2021-06-03

**Authors:** Lin-Hua Jiang, Elena Adinolfi, Sébastien Roger

**Affiliations:** ^1^ School of Biomedical Sciences, Faculty of Biological Sciences, University of Leeds, Leeds, United Kingdom; ^2^ Department of Medical Sciences, University of Ferrara, Ferrara, Italy; ^3^ EA4245, Transplantation, Immunology and Inflammation, Faculty of Medicine, University of Tours, Tours, France

**Keywords:** ion channels, cancer, cell death, proliferation, migration, metastasis, resistance to treatment

Mammalian cells express a large number of structurally distinct ion channels on the cell surface and also in the membranes of intracellular organelles. They can selectively permeate ion species (thus named Ca^2+^, Na^+^, K^+^ or Cl^−^ channels according to their selectivity) or a group of positively or negatively charged ions (non-selective cation or anion channels) (Hille, 2001). Their activity is regulated by diverse physical, biochemical or biological stimuli of the microenvironments and, as such, they initial or control important signaling mechanisms involved in a wide range of cellular processes which are critical for cancer development and progression including cell differentiation, proliferation, migration and apoptosis. It comes no surprise that an alteration in ion channel expression and/or activity is causatively associated with manifestation of cancer hallmarks (Hanahan and Weinberg, 2011), leading to the introduction of oncochannels (Huber, 2013).

This Research Topic collects 16 review and original research articles, which provide idea-provoking insights or new research regarding the role of ion channels in cancer and the concept or efforts of targeting them to treat cancer.

## Role of Ion Channels in Cancer Cells and Host-Tumour Cross-Talk

Intracellular Ca^2+^ homeostasis disruption, and aberrant activation of downstream signalling pathways is a well-established cause of cancerous transformation (Gross et al., 2020). Tajada and Villalobos summarized the expression profile of voltage-gated Ca^2+^ channels (Ca_V_1.2, Ca_V_1.3, Ca_V_1.4, Ca_V_2.1, Ca_V_2.2, Ca_V_3.1, Ca_V_3.2, Ca_V_3.3), transient receptor potential channels (TRPC1, TRPC3, TRPC4, TRPV4, TRPV6, TRPM6, TRPM7, TRPM8), and Ca^2+^ release activated Ca^2+^ or store-operated Ca^2+^ (CRAC/SOC) channels (ORAI1, ORAI3) in various cancer types. They also discussed the complex and opposing roles of these Ca^2+^ channels in regulating cancer cell proliferation, migration, invasion, and apoptosis resistance. Finally, they discussed the anti-cancer effects of the available Ca_V_ and CRAC channel modulators, non-steroidal anti-inflammatory drugs and polyamine biosynthesis inhibitors that directly or indirectly modulate the expression and/or activity of the CRAC channels.

Diaz-Garcia and Varela focused on the voltage-gated K^+^ (K_V_) and Na^+^ (Na_V_) channels in cancer cells. Several K_V_ channels, including K_V_1.1, K_V_1.3, K_V_10.1 [also known as the human ether-à-go-go (hEAG)], K_V_11.1 [also known as the human ether-àgo-go related (hERG) or hERG1] and K_Ca_1.1 (also known as the big-conductance Ca^2+^-activated K^+^ or BK_Ca_), are highly expressed in many cancer types, and their overexpression is linked with almost all known cancer hallmarks. Overexpression of the Na_V_ channels in multiple cancer types, either the pore-forming α-subunits or ancillary β-subunits, is also causatively associated with increased cell migration, invasion and metastasis, exemplified by the neonatal variant Na_V_1.5 (nNa_V_1.5) in breast and colorectal cancers, Na_V_1.6 in cervical and colorectal cancers, and Na_V_1.7 in prostate, lung and gastrointestinal tract cancers. They also highlighted that some scorpion venom toxins that inhibit the K_V_ (K_V_1.1, K_V_1.3 or BK) or Na_V_ channels (Na_V_1.6 or Na_V_β1) have been shown to produce anti-cancer effects, and could therefore be considered as valuable anti-cancer tools.

Schnipper et al. and Hofschröer et al. reviewed a variety of Ca^2+^, K^+^, Na^+^ and Cl^−^ channels involved in pancreatic ductal adenocarcinoma (PDAC). These ion channels include TRP channels (TRPC1, TRPV1, TRPV6, TRPM2, TRPM7, TRPM8), ORAI1 CRAC channel, and ATP-gated Ca^2+^-permeable channel P2X7 receptor; K_V_ channels (K_V_1.3, hEAG, hERG), intermediate Ca^2+^-activated K^+^ channel (IK_Ca_, also known as K_Ca_3.1), two-pore domain K^+^ (K_2P_) channels (TWIK-1, TREK-1, TASK-1); Na_V_ and acid-sensitive ion (ASIC1, ASIC3) channels; Ca^2+^-activated Cl^−^ channel TMEM16A and related proteins, and cystic fibrosis transmembrane regulator (CFTR). Altered expression of many of these channels, some being upregulated and others downregulated, is known to correlate with low overall patient survival. Hofschröer et al. also described the role of ion channels in the cross-talk between cancer, stroma, and immune cells in PDAC. Indeed, in this malignancy, ion channel activity not only affects cancer cell proliferation and invasion but also extracellular matrix remodeling and fibrosis together with tumour-infiltrating immune cell populations.

Restrepo-Angulo et al. examined the regulation of ion channel expression in cancer cells by sex steroid hormones, such as 17-β estradiol and dihydro-testosterone, and steroid pro-hormones like vitamin D and its active metabolite calcitriol. Exposure to 17-β estradiol, dihydro-testosterone or calcitriol regulated, mainly through a transcriptional mechanism, the expression of ion channels in breast (BK_Ca_, hEAG, nNav1.5, Ca_V_1.2, ORAI3, TRPV6, CLC-3), cervical (BK_Ca_, hEAG), endometrial (BK_Ca_, Ca_V_1.3) and prostate (TRPC6) cancers, with upregulation in most cases and downregulation in some cases. Ensuing gain or loss of the channel activity in many but not all cases led to increased cell proliferation, migration, invasion and/or tumor progression. The interesting exception is that calcitriol-induced downregulation of hEAG in breast cancer and TRPC6 in prostate cancer attenuated cell proliferation, suggesting calcitriol as a promising co-adjuvant for the treatment of vitamin D receptor-expressing cancers.

Fukushiro-Lopes et al. examined the role of the ATP-sensitive K^+^ (K_ATP_) channel made of inwardly-rectifying Kir6.2 pore-forming subunit and sulphonylurea receptor 2 (SUR2) regulatory subunit in gynecologic cancers. Treatment with minoxidil, an anti-hypertensive drug that activates the Kir6.2/SUR2 channel via binding to SUR2, prevented development and metastasis in xenogeneic murine models of ovarian carcinoma using human ovarian OVCAR-8 cancer cells. In OVCAR-8 cells and also in human endometrial SPEC-2 cancer cells, treatment with minoxidil resulted in inhibition of cell proliferation via attesting cell recycle, and also induction of apoptosis via Ca^2+^ influx-dependent mitochondrial dysfunction, mitochondrial ROS generation and oxidative DNA damage. These findings raise an attractive possibility of repurposing drugs activating the Kir6.2/SUR2 channel as a new or adjuvant therapeutic approach for ovarian carcinoma.

Palme et al. investigated the effect of ionizing radiation on hERG channel expression in human chronic myeloid leukemia (CML) cells and the implication to radiotherapy. Exposure to sub-lethal radiation induced an increase in the K^+^ currents that were sensitive by the hERG inhibitor E4301 in CML K562 cells and primary human CML cells. Treatment with E4301 resulted in a greater increase in the steady-state intracellular Ca^2+^ level in irradiated K562 cells than in control cells, but reduced the activation of Ca^2+^/calmodulin (CaM)-dependent kinase II. Importantly, treatment with E4301 prevented cell arrest and reduced clonogenic survival of irradiated cells, thus conferring radio-resistance.

As mentioned above, P2X7 is expressed in many cancer types, in stromal and immune cells of the tumor microenvironment (TME) as well as in cancer cells. Lara et al. provided a critical and in-depth analysis of the molecular and signaling mechanisms determining the complex and not yet fully understood role of P2X7 in cancer, or the receptor activity reported so far in different oncological conditions. They also discussed the possible strategies for developing anti-cancer drugs targeting P2X7 with either agonists that induce large pore formation to favor cancer cell death, or antagonists that would reduce P2X7-dependent oncogenic transformation. Chimote et al. analyzed the role of the Ca^2+^-activated K^+^ channel K_Ca_3.1 in tumor infiltration by cytotoxic lymphocytes. They demonstrated that K_Ca_3.1 is central in impaired chemotaxis of CD8^+^ cells toward the TME in head and neck cancer and that its function critically depends on the level of membrane-associated Ca^2+^ sensor CaM that was reduced in circulating lymphocytes of head and neck cancer patients. Interestingly, K_Ca_3.1 activation with 1-EBIO restored the chemotaxis and tumour-eradicating ability of cells and even amplified the anti-inflammatory response by increasing INFγ production, suggesting that K_Ca_3.1 antagonism could be a suitable therapeutic strategy in head and neck cancer.

## Interplays of Ion Channels With Other Signaling Mechanisms in Cancer Cells

Luo et al. provided an overview of the mechanisms by which nNa_V_1.5 in breast cancer potentiates cell migration, invasion and metastasis, revealed by *in vitro* and *in vivo* studies mainly using MDA-MB-231 metastatic breast cells. One important mechanism is that nNa_V_1.5 promotes H^+^ efflux via allosteric stimulation of the Na^+^/H^+^ exchanger isoform-1 (NHE-1) that creates an extracellular acidic microenvironment to facilitate proteinases to degrade ECM, favoring cell migration and invasion. They also discussed various agents that reduced cell migration and invasion via inhibiting the Nav1.5 expression and activity or altering related signaling pathways.

Iorio et al. reported an important role for NHE1 in mediating hERG-dependent regulation of colorectal cancer (CRC) cell migration, using HCT116 and HT29 cells. Growing cells on surface coated with ECM proteins, collagen I or fibronectin, resulted in intracellular alkalinisation, which was prevented by treatment with NHE-1 inhibitor amiloride, NHE-1-specific inhibitor cariporide or hERG inhibitor E4031, as well as with an β1 integrin-blocking antibody. Cell migration on collagen I-coated surface was also attenuated by treatment with cariporide or E4031. Furthermore, exposure to collagen I enhanced protein-protein interactions of β1 integrin, NHE-1 and hERG. These results suggest that β1 integrin-mediated adhesion of CRC cells onto ECM proteins stimulates β1 integrin, hERG and NHE-1 to form a signaling complex that facilitates hERG-dependent stimulation of NHE-1, leading to H^+^ efflux and intracellular alkalinisation to drive cell migration.

Rapetti-Mauss et al. presented an overview of the regulation of the Wnt/β-catenin signaling pathway by ion channels in various cancer types. On one hand, several ion channels including KCNQ1, hERG, CFTR and ClC-2, suppress the Wnt pathway. Consistently, reduction in the expression and/or activity of KCNQ1 in CRC and hepatocellular carcinoma or CFTR and ClC-2 in CRC was shown to stimulate the activity of the Wnt pathway and cancer progression, leading to the notion of these channels as tumor suppressors. On the other hand, overexpression of TRPC5 in CRC, TRPV4 in gastric cancer, TRPM4 in prostate cancer, TRPM8 in prostate and breast cancers, P2X7 in osteosarcoma, and ASIC1a in liver cancer promotes the activity of the Wnt pathway, thereby inducing tumor growth, cell proliferation, migration, invasion, metastasis, and/or chemo-resistance.

Chinigò et al. reviewed the literature regarding bidirectional communications between the TRP channels and small GTPases in various cancer types. The TRP channels were shown to modulate the activity of small GTPase and related signaling pathways in a Ca^2+^-dependent or independent manner and, conversely, the small GTPases regulate the surface expression or gating of TRP channels. Such interplays were documented to occur between TRPC1, TRPC5, TRPC6, TRPV4, TRPM4 or TRPM7 and Rho GTPases in cell migration, between TRPV2 or TRPM2 and Rho GTPases in cell invasion, and between TRPC1, TRPC6, TRPV4 or TRPM8 and Rho/Ras GTPases in tumor vascularization.

## Targeting Oncochannels for Developing Therapeutic Tools

Recent years have witnessed increasing interest in targeting ion channels with new small molecules and repurposing existing drugs for cancer therapy. For example, Fukushiro-Lopes et al. proposed the Kir6/SUR2 channel activator minoxidil for the treatment of gynecologic cancer. In this vein, Leslie et al. examined the inhibitory effects on nNa_V_1.5 of eslicarbazepine acetate and slicarbazepine, the major circulating active metabolite and also the main enantiomer responsible for anticonvulsant activity are known to inhibit neuronal non-Na_V_1.5 channels. Both compounds inhibited nNa_V_1.5 endogenously expressed in MDA-MB-231 cells and Na_V_1.5 heterologously expressed in HEK293 cells, leading to the proposal of using such agents to treat metastatic breast cancer.

There are ongoing efforts of developing small molecular chemicals and biologics targeting particular ion channels as research tools to better understand their role in cancers and validate the feasibility of such interventions as cancer therapy. Demeules et al. discussed adeno-associated viral vectors producing P2X7-modfying nanobodies termed AAVnano, and demonstrated the advantage of such an AAVnano to intervene P2X7-mediated cell function or tumor growth in mice. Hartung et al. reported a nanobody against hEAG fused to an apoptosis-inducing ligand and showed its effectiveness in inducing apoptosis and inhibiting spheroid growth using hEAG-expressing human prostate DU-145 cancer cells. These studies highlight the potential of nanobodies, particularly the ones combining cancer specificity and cytotoxicity, as a new approach for cancer therapy.

In summary, studies continue to accumulate the evidence supporting a vital role of an alteration in the expression and/or activity of ion channels in both cancer cells and cancer-contacting cells in determining cancer hallmarks. As such, altered ion channel expression or activity has been proposed by many researchers as a cancer hallmark. Targeting onco-channels has become an attractive strategy of developing novel anti-cancer treatment.
